# Maximizing Terahertz Energy Absorption with MXene Absorber

**DOI:** 10.1007/s40820-023-01167-6

**Published:** 2023-08-12

**Authors:** Xinliang Li, Hao Luo

**Affiliations:** 1 Key Laboratory of Material Physics, Ministry of Education, School of Physics and Microelectronics, Zhengzhou University, Zhengzhou, 450052 People’s Republic of China; 2 School of Materials Science and Engineering, Zhengzhou University, Zhengzhou, 450052 People’s Republic of China

**Keywords:** Terahertz absorption, MXene absorber, Impedance matching, Electron concentration, Relaxation time

## Abstract

Achieving high absorption in broad terahertz bands has long been challenging for terahertz electromagnetic wave absorbers. Recently in Nature Photonics, Xiao et al. reported the high absorption approaching the theoretical upper limit across the whole terahertz band of MXene-based terahertz absorbers and, on this basis, constructed an applicable, updated alternating current impedance matching model.

**Graphical Abstract:**

## Highlights

- MXene film absorbers enable the near theoretical absorption strength in extremely wide wavebands of 0.5–10 THz.

## Graphical abstract

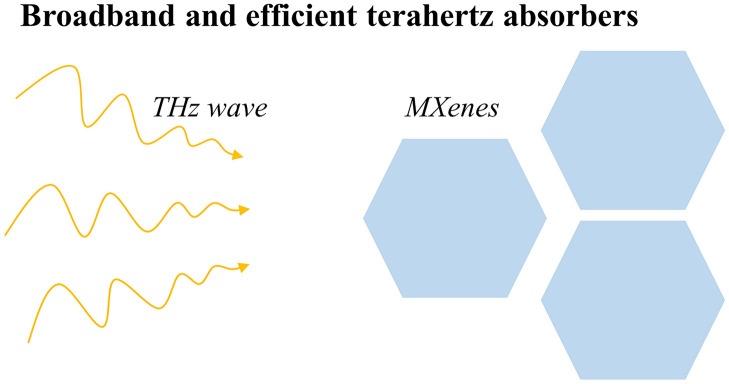

Advances in electronic communications and imaging applications are contingent on innovations in terahertz (THz) electromagnetic wave absorption technology. THz absorbers are evaluated based on two decisive parameters: absorption efficiency and bandwidth, both of which researchers aim to maximize [1]. The well-established electromagnetic theory posits that electron concentration and relaxation time are the key determinants for achieving high absorption in a broad THz band. In particular, the ideal impedance matching case predicts an upper limit of absorption efficiency at 50%, where the square resistance of absorbers is half of the free space impedance (Z_o_/2) [2]. Additionally, an essential criterion for realizing effective bandwidth coverage of the entire THz band is a relaxation time of free electrons that is less than 15 fs. Nevertheless, documented facts show that absorbers developed based on metals, graphene, and topological insulators generally achieve high absorption only within a narrow range of THz bands rather than across the desired broadband. Consequently, current researchers have concentrated their efforts on screening a broad range of candidates to address the enduring issue of narrow effective absorption THz band under the guidance of the classical direct current (d.c.) impedance matching model.

Recently, Xiao and coworkers from University of Electronic Science and Technology of China reported an application-oriented high-efficiency THz absorber based on two-dimensional transition metal carbide film, specifically Ti_3_C_2_T_*x*_ MXene [3]. This thin-film absorber exhibited remarkable absorption efficiency approaching the theoretical upper limit in the ultra-broad THz band. With outstanding intrinsic properties in electron concentration (~ 1021 cm^−3^) and relaxation time (< 13 fs), 10.2-nm-thick Ti_3_C_2_T_*x*_ MXene film synthesized by the topochemical deintercalation method exhibited nearly 50% THz absorption in the frequency band of 0.5–10 THz. More importantly, they validated that alternating current (a.c.) impedance matching model rather than classic d.c. impedance matching model is more suitable to explain the strong interaction between MXene and THz electromagnetic waves.

Their study reveals that the thickness factor of Ti_3_C_2_T_*x*_ MXene absorbers significantly influences absorption efficiency and effective bandwidth (Fig. 1a). Ti_3_C_2_T_*x*_ MXene film with a thickness of only 3 nm can achieve decent absorption efficiency of over 30% in the 0.5–4.5 THz band. Increasing the thickness to 10.2 nm optimizes the absorption performance to nearly 50%. However, further increasing the thickness results in a counterproductive trend. For example, at 13.3 nm, the average absorption efficiency was determined to be about 46%. Meanwhile, the fluctuation of absorption efficiency displays the same trend. It varies within 2% with thickness and bottoms out at 10.2 nm to 0.108% (Fig. 1b). Note that when the frequency band is extended to 10 THz, the 10.2 nm Ti_3_C_2_T_*x*_ MXene film still shows similar high absorption and always remains stable. The underlying mechanism of this trade-off is attributed to the fact that stacking of more Ti_3_C_2_T_*x*_ MXene flakes leads to higher surface electron enrichment, continuously reducing the square resistance. The entire evolution process experiences the approach and deviation of the ideal impedance matching case.

**Fig. 1:**
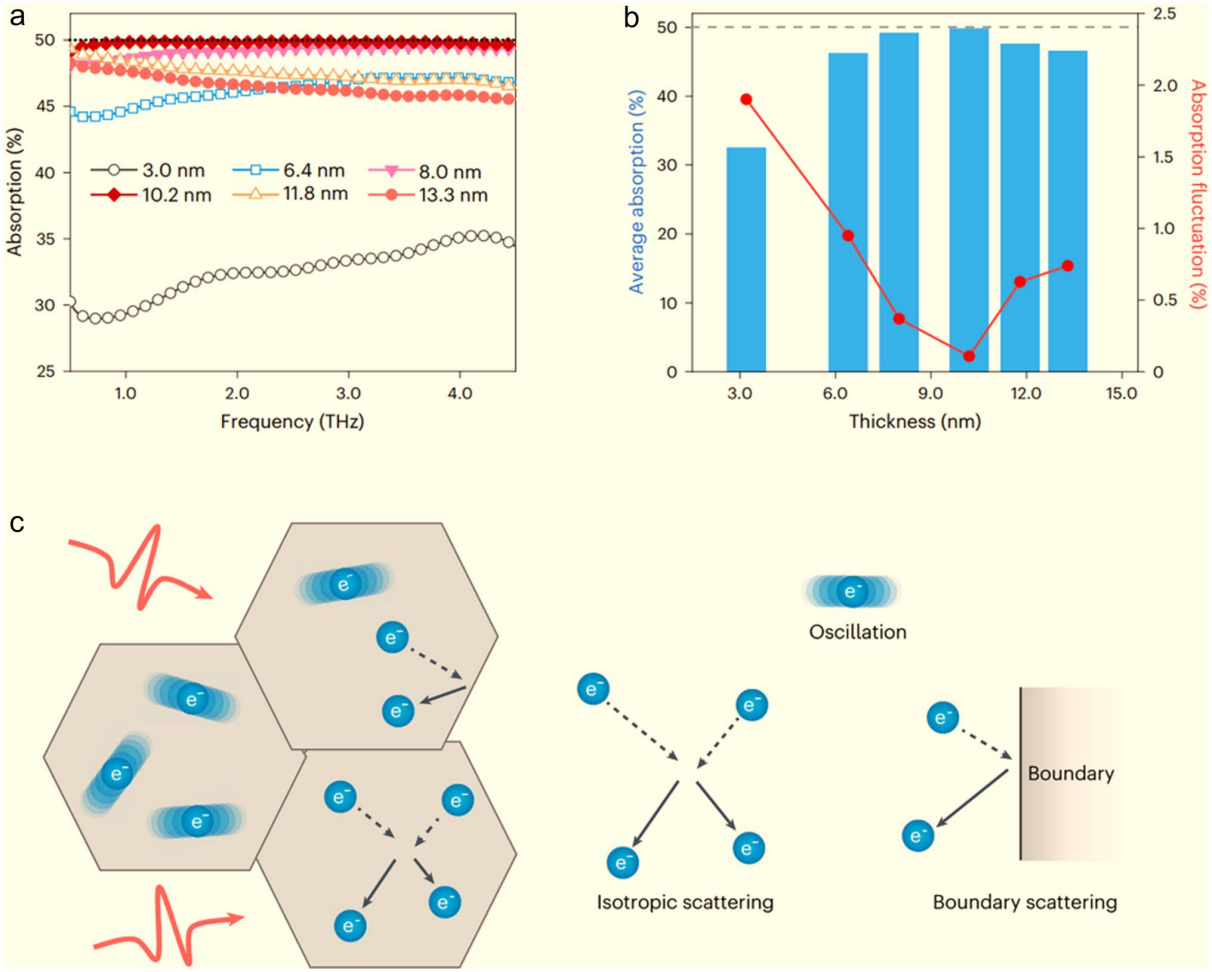
**a** Terahertz absorption properties of Ti_3_C_2_T_*x*_ assemblies with different thicknesses for ~ 0.5–4.5 THz. **b** Average absorption and absorption fluctuation of Ti_3_C_2_T_*x*_ assemblies with different thicknesses for ~ 0.5–4.5 THz. **c** Schematic of the charge transport model in Ti_3_C_2_T_*x*_ MXene assemblies under terahertz radiation. Copyright 2023, Springer Nature

The reported efficient broadband absorption of Ti_3_C_2_T_*x*_ MXene absorbers poses a challenge to the classic d.c. impedance model. In the optimal performance case, the square resistance (6.5 kΩ □^−1^) is an order of magnitude higher than the theoretical value (*Z*_o_/2). To address this issue, Xiao and coworkers construct an equivalent a.c. impedance model based on measured THz conductivity, which perfectly matches the *Z*_o_/2 value and supports the experimental data. The authors attribute the huge dielectric difference of Ti_3_C_2_T_*x*_ MXene absorbers under the two modes to their complex electron conduction pathway (Fig. 1c). When incident THz electromagnetic waves encounter Ti_3_C_2_T_*x*_ MXene flakes, most free electrons tend to conduct in the interior of the flakes due to their larger lateral size (microns) compared to the diffusion length of electrons (tens of nanometers) [4]. A few electrons scatter and hop between flakes when encountering lattice defects and flake boundaries. Therefore, THz conduction is dominated by intraflake electron transport. The backscattering behavior has a distinct size effect, in which the small flake size and rich boundaries intensify the scattering possibility. In practice, however, d.c. measurements require electrode spacing well beyond the lateral size of a single Ti_3_C_2_T_*x*_ MXene flake, resulting in interflake transport model of electrons dominating the THz conduction. This sharp deviation renders the d.c. impedance model inapplicable to Ti_3_C_2_T_*x*_ MXene absorbers, except for the case of unbounded and low-frequency bands, where the difference between a.c. and d.c. impedance models is not significant [5].

The Drude-Smith model effectively reveals the interaction between MXene and THz electromagnetic waves [6]. The derived relaxation-time-dependent conductivity curve indicates that reducing relaxation time benefits the extension of effective absorption bandwidth. Moreover, the relaxation time is negatively correlated with the fluctuation of THz conductivity, which means that a short relaxation time will result in stable THz absorption in the broadband. Furthermore, electron concentration plays a crucial role in the effective MXene-THz electromagnetic wave interaction. It is a function of impedance matching and can be precisely adjusted by modulating flake size and thickness. Inappropriate electron concentration will lead to impedance mismatching, which deteriorates absorption efficiency and effective bandwidth. The study concludes that the intrinsic compatibility of high electron concentration and short relaxation time is the essential driving force for Ti_3_C_2_T_*x*_ MXene film to achieve the absorption limit in the ultra-broad THz band.

Overall, Xiao and coworkers have demonstrated the exceptional absorption performance of Ti_3_C_2_T_*x*_ MXene in the THz frequency range. The fabrication of thin-film MXene absorbers is cost-effective, simple, and easily scalable. This work marks a significant advancement in THz detection technology with high absorption efficiency and broad effective bandwidth. The constructed a.c. impedance matching model significantly improves the existing theoretical framework for designing high-performance THz absorbers, which can benefit both experts and novices in the field. Nevertheless, since few-flake MXenes are highly sensitive to light and heat, future studies on MXene absorbers should focus on dynamic structural and phase changes during their long-term service [7].

## References

[1] Yao B, Liu Y, Huang S, Choi C, Xie Z (2018). Broadband gate-tunable terahertz plasmons in graphene heterostructures. Nat. Photonics.

[2] Pham P, Zhang W, Quach N, Li J, Zhou W (2017). Broadband impedance match to two-dimensional materials in the terahertz domain. Nat. Commun..

[3] Zhao T, Xie P, Wan H, Ding T, Liu M (2023). Ultrathin MXene assemblies approach the intrinsic absorption limit in the 0.5–10 THz band. Nat. Photonics.

[4] Li X, Li M, Li X, Fan X, Zhi C (2022). Low Infrared emissivity and strong stealth of Ti-based MXenes. Research.

[5] Li G, Kushnir K, Dong Y, Chertopalov S, Rao A (2018). Equilibrium and non-equilibrium free carrier dynamics in 2D Ti_3_C_2_T_x_ MXenes THz spectroscopy study. 2D Mater..

[6] Cocker T, Baillie D, Buruma M, Titova L, Sydora R (2017). Microscopic origin of the Drude-Smith model. Phys. Rev. B.

[7] Li X, Huang Z, Shuck C, Liang G, Gogotsi Y (2022). MXene chemistry, electrochemistry and energy storage applications. Nat. Rev. Chem..

